# A natural history study of autosomal dominant *GUCY2D*-associated cone–rod dystrophy

**DOI:** 10.1007/s10633-023-09954-7

**Published:** 2023-09-29

**Authors:** Amanda J. Scopelliti, Robyn V. Jamieson, Elizabeth H. Barnes, Benjamin Nash, Sulekha Rajagopalan, Elisa L. Cornish, John R. Grigg

**Affiliations:** 1 Save Sight Institute, Specialty of Clinical Ophthalmology and Eye Health, Faculty of Medicine and Health, The University of Sydney, Sydney, NSW Australia; 2 Eye Genetics Research Unit, Sydney Children’s Hospitals Network, Save Sight Institute, Children’s Medical Research Institute, The University of Sydney, Sydney, NSW Australia; 3 NHMRC Clinical Trials Centre, Faculty of Medicine and Health, University of Sydney, Sydney, NSW Australia; 4 Sydney Genome Diagnostics, Western Sydney Genetics Program, Sydney Children’s Hospitals Network, Sydney, NSW Australia; 5 Department of Clinical Genetics, Liverpool Hospital, Locked Bag 7103, Liverpool, NSW Australia

**Keywords:** GUCY2D, Cone–rod dystrophy, Inherited retinal disease, Electroretinography, Autosomal dominant, Retinal guanylate cylase-1

## Abstract

**Purpose:**

To describe the natural history of autosomal dominant (AD) GUCY2D-associated cone–rod dystrophies (CRDs), and evaluate associated structural and functional biomarkers.

**Methods:**

Retrospective analysis was conducted on 16 patients with AD GUCY2D-CRDs across two sites. Assessments included central macular thickness (CMT) and length of disruption to the ellipsoid zone (EZ) via optical coherence tomography (OCT), electroretinography (ERG) parameters, best corrected visual acuity (BCVA), and fundus autofluorescence (FAF).

**Results:**

At first visit, with a mean age of 30 years (range 5–70 years), 12 patients had a BCVA below Australian driving standard (LogMAR ≥ 0.3 bilaterally), and 1 patient was legally blind (LogMAR ≥ 1). Longitudinal analysis demonstrated a deterioration of LogMAR by − 0.019 per year (*p* < 0.001). This accompanied a reduction in CMT of − 1.4 µm per year (*p* < 0.0001), lengthened EZ disruption by 42 µm per year (*p* =  < 0.0001) and increased area of FAF by 0.05 mm^2^ per year (*p* = 0.027). Similarly, cone function decreased with increasing age, as demonstrated by decreasing b-wave amplitude of the light-adapted 30 Hz flicker and fused flicker (*p* = 0.005 and *p* = 0.018, respectively). Reduction in CMT and increased EZ disruption on OCT were associated with functional changes including poorer BCVA and decreased cone function on ERG.

**Conclusion:**

We have described the natural long-term decline in vision and cone function associated with mutations in GUCY2D and identified a set of functional and structural biomarkers that may be useful as outcome parameters for future therapeutic clinical trials.

**Supplementary Information:**

The online version contains supplementary material available at 10.1007/s10633-023-09954-7.

## Introduction

Cone (CD) and Cone Rod dystrophies (CRD) are a group of inherited retinal dystrophies (IRD) that are characterised by the degeneration of the photopic (cone) system. The phenotypic naming of the condition is based on the patient symptoms and visual electrophysiology findings. Patients with Cone dystrophy usually present in adolescence or early adulthood with decreased visual acuity, photophobia, and dyschromatopsia [1, 2]. In many cases patients will progress, developing nyctalopia and loss in peripheral vision at low light levels due to rod degeneration, thereby conferring a subsequent diagnosis of cone–rod dystrophy (CRD) [1]. Several genes associated with CRD have been described that display autosomal recessive (AR), autosomal dominant (AD) and X-linked patterns of inheritance, as listed on RetNet, the University of Texas School of Public Health Retinal Information Network [3]. Many of the genes identified have been associated with multiple phenotypes, or disease in multiple inheritance patterns, with a subgroup associated with multiple phenotypes in multiple inheritance patterns.

One member of this subgroup is the *GUCY2D* gene, which encodes the photoreceptor guanylate cyclase (GC-E). GC-E is a membrane-bound protein that restores photoreceptor cGMP levels following the light-induced signal cascade, and is indirectly regulated by Ca^2+^ levels. More than 140 disease-causing GUCY2D mutations have been identified, of which 88% are associated with AR Leber congenital amaurosis (LCA) and 9% with AD CD/CRD [4]. Recently a further phenotype described as congenital night blindness has been associated with biallelic GUCY2D variants [5]. The mutations associated with AD CD/CRD are largely confined to exon 13 of GUCY2D, which encodes the GC-E dimerisation domain, with a hotspot at Arg838. In vitro [6] and in vivo [7] studies have shown that Arg838 mutations generate a functional cyclase with reduced sensitivity to Ca^2+^-mediated activation. In contrast, the mutations associated with LCA are found scattered throughout the gene [4], and generate GC-E with impaired catalytic ability [8] or misfolded protein that is subsequently degraded [9].

Recently, results from a Phase I/II human clinical trial of subretinal gene therapy for AR GUCY2D-LCA were published [10], demonstrating early indication of safety and efficacy of the treatment. Since variants in AD GUCY2D lead to a presumed dominant-negative disease mechanism, gene editing strategies have been tested in mouse and macaque as a therapeutic approach [11]. To prepare for potential therapeutic interventions for AD GUCY2D-CRD, it is imperative that outcome measures are identified that can reliably monitor disease progression and patient visual function. Two recently published studies described cohorts from Europe and the United Kingdom with GUCY2D-CRD, highlighting disruption of the ellipsoid zone (EZ) and visual acuity (VA) as two important endpoints for future therapeutic trials [12, 13]. Our study describes an Australian cohort of AD GUCY2D-CRD patients, highlighting key functional and structural biomarkers and their change with disease duration, providing further insights into disease progression.

## Methods

The medical records of 16 patients with disease-causing variants of *GUCY2D* attending two tertiary referral centres (Save Sight Institute, Sydney, NSW, Australia, Sydney Eye Hospital Campus, Sydney, NSW, Australia and Sydney Children’s Hospitals Network, Sydney, NSW, Australia) between January 2003 and January 2021 were reviewed. Molecular diagnoses were achieved via genetic testing performed through National Association of Testing Authorities-accredited genetic laboratories. The study was approved by the Local Ethics Committee and adhered to the tenets of the Declaration of Helsinki.

All available medical records were reviewed and clinical data including patient age, gender and best corrected visual acuity (BCVA) were recorded from all visits. Visual acuity was measured using the Early Treatment Diabetic Retinopathy Study Chart [14], and letter acuity was recorded using metric scale, as per standard clinical recordings. To assist with data analysis, all BCVA values are expressed as logarithm of minimum angle of resolution (logMAR) notation.

**Fig. 1:**
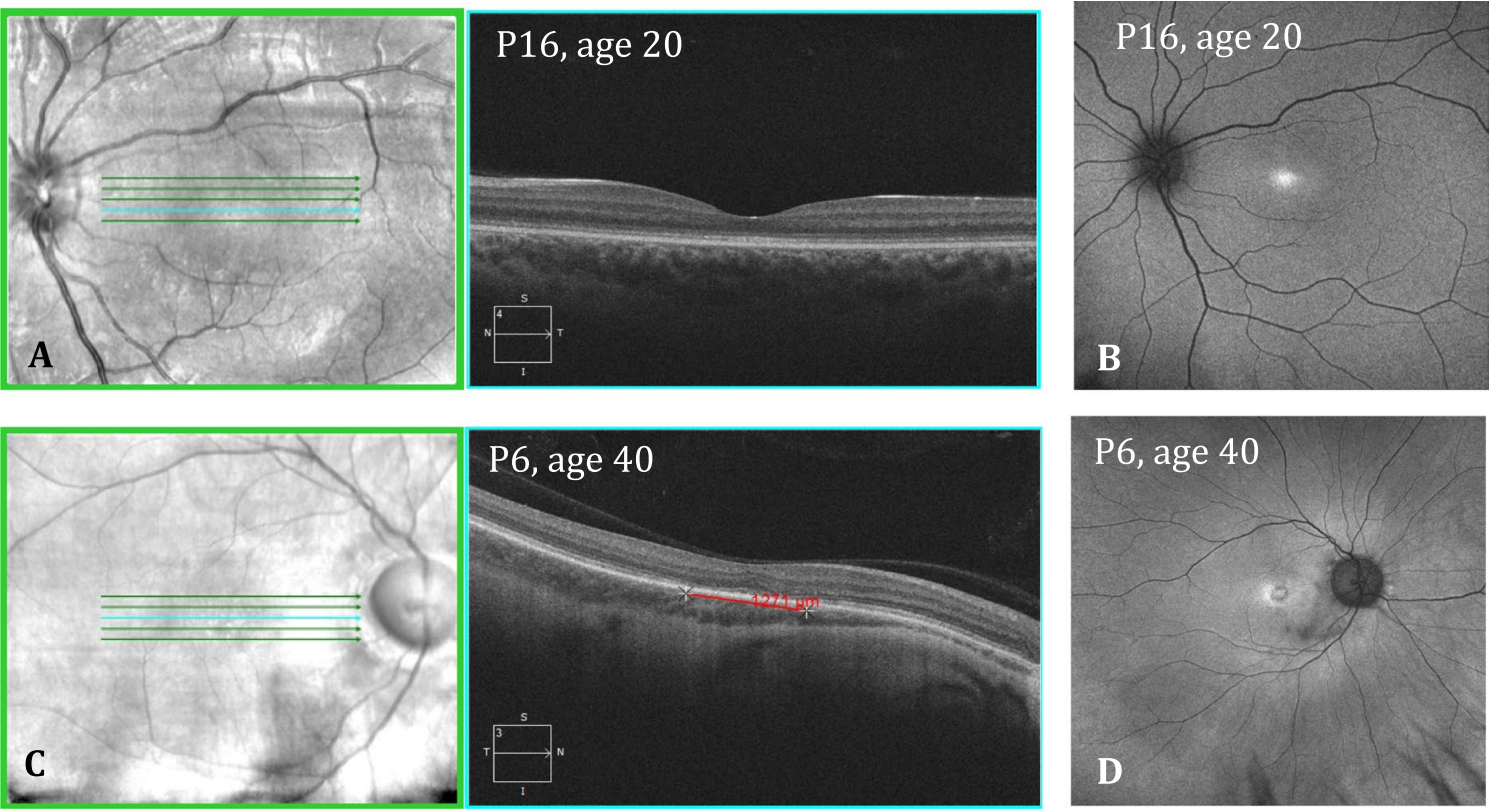
Sample of findings on imaging in patients with GUCY2D-associated CRD. For patient P16 (R383C) at 20 years old, **A** depicts SD-OCT scan, showing a continuous ellipsoid zone and (**B**) shows FAF image with foveal hyperautofluorescence. BCVA was 0.18 logMAR in both eyes. For patient P6 (R383H) at 40 years old, **C** depicts SD-OCT scan, showing a disruption of ellipsoid zone 1271 µm long and (**B**) shows FAF image with foveal hyperautofluorescence in a bull’s eye pattern. BCVA was 0.90 logMAR in both eyes

Imaging was also collected, including ultra-widefield fundus autofluorescence (UW-FAF) and spectral domain optical coherence tomography (SD-OCT). UW-FAF imaging was obtained using Optos California (Optos plc, Dunfermline, UK) encompassing up to 200° (532 nm excitation) (Fig. 1). Dimensions of hyperautofluorescent areas were measured using the caliper tool embedded into the UW-FAF software. SD-OCT scans of the macula were captured using the Spectralis SD-OCT (Heidelberg Engineering, Heidelberg, Baden-Württemberg, Germany) or Cirrus SD-OCT (Carl Zeiss Meditec, Dublin, California, United States). Poor quality images were rejected at the time of examination, as per standard protocols. Manual review and correction of segmentation for all macular volume scans was performed to ensure quality control. Average macular thickness (AMT) was calculated from the nine areas depicted in the Early Treatment Diabetic Retinopathy Study (ETDRS) [14] map generated by the SD-OCT software. Integrity of the ellipsoid zone (EZ) was assessed via high definition cross-sectional scans through the macula, and the caliper tool embedded within the SD-OCT software was used to measure the length of disruption of the EZ (Fig. 1). Cirrus SD-OCT values were corrected using conversion tables and made comparable to those collected with Spectralis OCT in order to minimize the thickness differences existing between the two instruments [15].

Results from International Society for Clinical Electrophysiology standard full-field electroretinogram (ffERG) and pattern electroretinogram (pERG) results were collected [16]. The ffERG parameters analyzed include light-adapted 30 Hz flicker amplitude (µV) and peak time (ms), and fused flicker amplitude (µV). The fused flicker is a non-ISCEV standard waveform that enables assessment of residual cone function, as described by Berson. The flicker fusion ERG is the amplitude of the fundamental of the ISCEV 30 Hz flicker ERG, produced by narrow band filtering after averaging (i.e. not Fourier techniques post hoc.) [17, 18]. The pERG parameters analyzed include P50 amplitude (µV) and N95 amplitude (µV) to a 15° field.

Statistical analyses were performed using SAS version 9.4. Continuous data were reported as mean ± standard deviation (SD), range and quartiles as appropriate. Categorical data were expressed in proportions. To account for correlation between repeated measurements from the same individual, structure–function relationships were analyzed using generalised estimating equations (GEE) assuming a normal distribution of the outcome and exchangeable correlation structure, from which we report the model co-efficient β.

## Results

16 patients (32 eyes) with CRD and *GUCY2D* mutations were identified (Table 1). No patients with confirmed *GUCY2D* mutations were excluded. There were six (37.5%) males and ten females (62.5%) from six families, with a mean age of 30 years at first presentation to the clinics (range = 5–70 years). The length of follow-up ranged from 0 to 17 years (mean ± standard deviation [SD], 7 ± 6.18 years).

**Table 1:** Patient demographics at first visit, or first measurement

Patient	Family	Sex	Age yrs	F/U yrs	Mutation	Eye	BCVA LogMAR	AMTμm	EZ disruption μm	LA 30 Hz amplitude μV	FAF mm
1	A	M	52	11	R838H	OD	0.88	231	1468	35	1.9
						OS	1.3	225	1596	37	2.1
2	A	M	19	9	R838H	OD	0.3	285^a^	0^a^	75	1
						OS	0.18	285^a^	0^a^	59	1
3	A	M	26	7	R838H	OD	0.48	270	1195	55	1.1
						OS	0.48	269	1022	73	1.2
4	B	M	29	11	R838H	OD	0.88	252^b^	0	38	1
						OS	0.4	249^b^	0	48	1.3
5	C	F	46	6	R838C	OD	0.3	246	0	50	0.8
						OS	0.3	243	0	62	0.8
6	B	F	28	13	R838H	OD	0.48	228^c^	1555^c^	42	1.7^c^
						OS	0.54	231^c^	1746^c^	53	1.9^c^
7	D	M	23	< 1	R838C	OD	0.6	243	995	33	1.2
						OS	0.6	242	1085	33	1.3
8	E	M	31	17	R838C	OD	0.7	245^d^	1494^d^	34^e^	1.9^d^
						OS	0.88	244^d^	2115^d^	32^e^	1.8^d^
9	E	F	6	12	R838C	OD	0.48	239^f^	0^f^	34	0^f^
						OS	0.48	237^f^	0^f^	33	0^f^
10	F	F	35	< 1	R838H	OD	0.48	247	518	28	0.8
						OS	0.48	238	393	28	1.2
11	F	F	10	< 1	R838H	OD	0.18	272	0	29	0.7
						OS	0.1	276	0	28	0.7
12	F	F	70	< 1	R838H	OD	1.48	207	2782	36	3
						OS	1.48	213	2577	x	2.5
13	F	F	43	< 1	R838H	OD	0.6	242	954	30	1
						OS	1	246	771	30	0.8
14	F	F	20	< 1	R838H	OD	0.18	276	0	28	3.1
						OS	0.0	273	0	27	3.4
15	G	F	40	12	R838C	OD	0.48	229^ g^	667^ g^	-	0
						OS	0.54	229^ g^	737^ g^	19	0
16	G	F	7	13	R838C	OD	0.18	284^ h^	0^ h^	30	0
						OS	0.3	280^ h^	0^ h^	30	0

*F/U* years of follow-up*, BCVA* best corrected visual acuity, *AMT* average macula thickness*, LA* light-adapted*, EZ* ellipsoid zone*, FAF* fundus autofluorescence diameter, *x* erroneous measurement excluded,—data not available. For data that was not available at first visit, the first measurement is presented

^a^Age: 20, ^b^Age: 38, ^c^Age: 39, ^d^Age: 40, e^a^ge: 32, ^f^Age: 10, ^g^Age: 45, ^h^age: 12

**Fig. 2:**
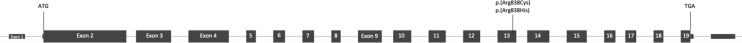
GUCY2D gene structure with variants identified in this study labelled above the affected exon

All patients in this cohort had disease-causing variants identified in *GUCY2D*. The variants identified were exclusively at amino acid position 838 (Fig. 2), which has previously been identified as a hotspot for mutation causing CRD. Ten patients from three families were heterozygous for p.Arg838His, and three patients from two families were heterozygous for p. Arg838Cys. Both of these variants have been previously associated with AD CRD [19–25]. One patient (P6) had an additional mutation in ABCA4 p.Gly1961Glu.

### Visual acuity

**Fig. 3:**
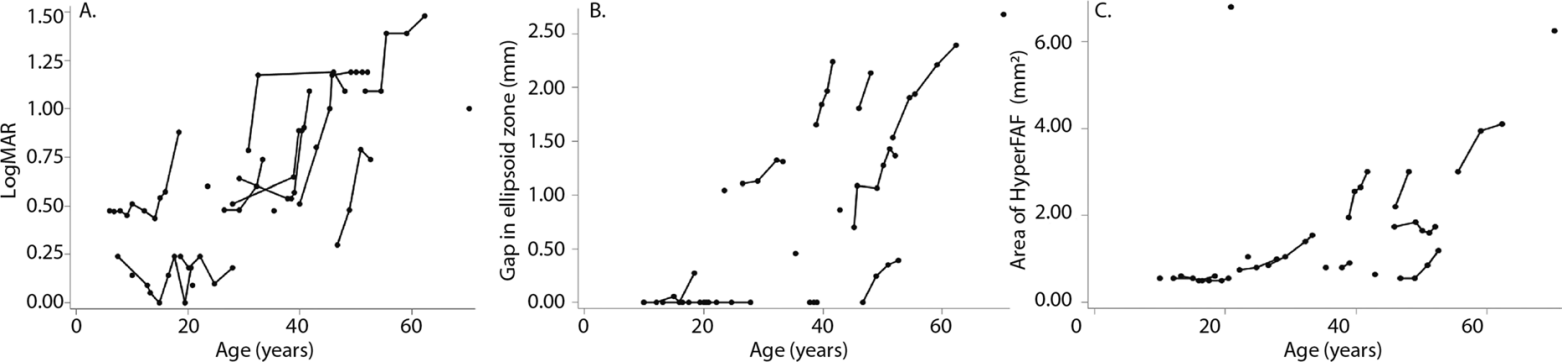
Worsening BCVA (**A**), lengthening of the disruption of ellipsoid zone (**B**) and increasing area of fundus hyperautofluorescence (**C**) are shown to progress with increasing age. Data is averaged over both eyes and lines shown depict the timeline of individual patients

Mean BCVA at first visit was 0.6 ± 0.4 LogMAR, with a range of 0–1.48 (32 eyes) (Table 2). 1/16 patient (P12) met the Australian definition of legal blindness (LogMAR ≥ 1 in both eyes) [26] at first visit, with a BCVA of 1.48 LogMAR in each eye. Three additional patients crossed the threshold of legal blindness during the observation period (P1, P8, P15) (Fig. 3A). The BCVA of 12/16 patients (75%) were below the Medical standards for an Australian Driver’s licence at first visit (LogMAR > 0.3 in both eyes) [27] (Table 1) with the BCVA of the 4 remaining patients remaining above this standard during the observation period (P2, P11, P14, P16) (Fig. 3A). Better visual acuity was associated with a larger ffERG LA 30 Hz b-wave amplitude (β = − 40 µV per 1 LogMAR, *p* =  < 0.0001; Table 3, Supplementary 1A) and fused flicker b-wave amplitude (β = − 13 µV per 1 LogMAR, *p* =  < 0.0001; Table 3, Supplementary 1C), however the association was lost when correcting for age (*P* = 0.24 and *P* = 0.77, respectively; Supplementary 5). Additionally, there was no association with peak time (β = − 5 µV per 1 LogMAR, *p* = 0.22; Table 3, Supplementary 1B). Association was found between BCVA and pERG N95 (β = − 1.1 µV per 1 LogMAR, *p* = 0.004; Table 3, Supplementary 1E), however this relationship was shown to interact with age (*p* = 0.006; Supplementary 5). No association was found between BCVA and pERG P50 (β = − 0.18 per µV*; p* = 0.50; Table 3, Supplementary 1D).

**Table 2:** Descriptive statistics

Variable	Mean	SD	Minimum	Maximum
BCVA (LogMAR)	0.62	0.41	0	1.48
Ellipsoid gap (µm)	849	904	0	2782
CMT (mm)	184	38	121	255
AMT (mm)	246	23	200	291
Area of hyper FAF (mm^2^)	1.6	1.5	0.4	7.4
LA 30Hz b-wave amplitude (µV)	50	27	2	152
LA 30Hz b-wave delay (ms)	32	5	26	55
Fused flicker b-wave amplitude (µV)	13	12	1	48
P50 (µV)	1.3	0.6	0.4	2.7
N95 (µV)	1.6	1.0	0.4	4.3

**Table 3:** Relationship between both ffERG and pERG electrophysiology parameters and BCVA, ellipsoid zone disruption, AMT and area of hyperautofluorescence. Model co-efficient β is expressed from GEE analysis, with 95% confidence intervals

Dependent variable	BCVA		Ellipsoid zone disruption	
	β-coefficient (95% CI)	*p*-value	β-coefficient (95% CI)	*p*-value
LA 30Hz b-wave amplitude (µV)	− 40.0 (− 59.5, − 20.6)	< 0.0001	− 18.9 (− 27.1, − 10.7)	< 0.0001
LA 30Hz b-wave delay (ms)	− 4.8 (− 12.5, 2.9)	0.22	2.5 (0.6, 4.5)	0.0096
Fused flicker b-wave amplitude (µV)	− 12.7 (− 19.3, − 6.2)	0.0001	− 4.4 (− 7.3, -1.5)	0.0028
P50 (µV)	− 0.2 (− 0.7, 0.4)	0.50	− 0.01 (− 0.29, 0.27)	0.93
N95 (µV)	− 1.1 (− 1.8, − 0.35)	0.004	0.2 (− 0.3, 0.7)	0.49

Dependent variable	AMT		Area of hyper FAF	
	β-coefficient (95% CI)	*p*-value	β-coefficient (95% CI)	*p*-value
LA 30Hz b-wave amplitude (µV)	0.7 (0.3, 1.1)	0.0016	− 3.3 (− 10.0, 3.4)	0.30
LA 30Hz b-wave delay (ms)	− 0.08 (− 0.15, 0.002)	0.058	0.6 (− 0.6, 1.9)	0.33
Fused flicker b-wave amplitude (µV)	0.3 (0.1, 0.4)	< 0.0001	1.1 (− 2.3, 4.5)	0.52
P50 (µV)	0.0003 (− 0.010, 0.011)	0.96	0.15 (0.06, 0.24)	0.0008
N95 (µV)	− 0.011 (− 0.029 to 0.007)	0.23	− 0.20 (− 0.43, 0.04)	0.097

Increasing age was associated with decreased visual acuity (β = 0.019 per year, *p* =  < 0.0001) (Fig. 3A). However, two patients demonstrated no deterioration of vision over time (P2 and P16, age 28 and 20 years at last visit, respectively). The rate of vision loss is not linear as highlighted in Fig. 3A, with the rate of vision loss more marked after the first and second decade of life. Increasing age was also associated with structural changes in the retina, including larger gap in the ellipsoid zone (β = 42 µm per year, *p* =  < 0.0001; Fig. 3B), decreasing central (β = -1.5 µm per year; *p* =  < 0.0001) and average (β = − 1.1 µm per year, *p* =  < 0.0001; Supplementary 2A) macula thickness, and increasing area of fundus autofluorescence (β = 0.05 mm^2^ per year, *p* = 0.027; Fig. 3C).

## Visual electrophysiology

**Fig. 4:**
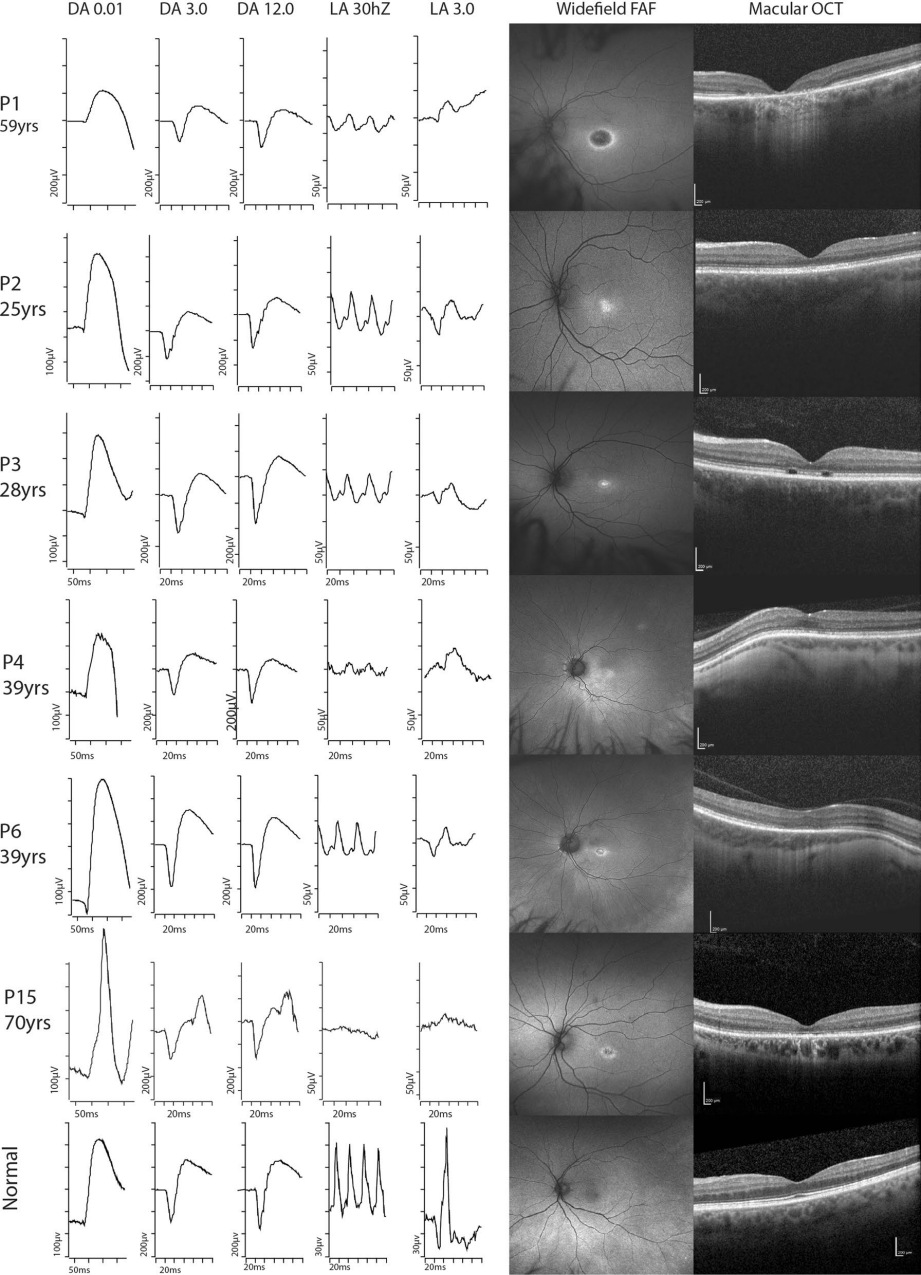
Representative electrophysiology and multimodal imaging in *GUCY2D* retinopathy

Cone function was also demonstrated to decrease with increasing age, with the amplitude of ffERG light-adapted (LA) 30 Hz flicker and fused flicker decreasing over time (β = − 0.8 µV per year, *p* = 0.005; β = − 0.2 µV per year, *p* = 0.018, respectively; Supplementary 2B and 2D). However, no evidence of age-related delay was observed for the ffERG LA 30 Hz peak time (β = 0.09 µV per year, *p* = 0.10; Supplementary 2C), and no association between age and the amplitude of pERG P50 and N95 parameters (β = 0.0001 µV per year, *p* = 0.98; β = 0.01 µV per year, *p* = 0.25; Supplementary 2E and 2F). Representative ISCEV standard ffERG, wide field fundus autofluorescence and OCT scans are presented in Fig. 4 which illustrates the variability in both the photopic ffERG responses and the multimodal imagining.

### Optical coherence tomography (OCT)

**Fig. 5:**
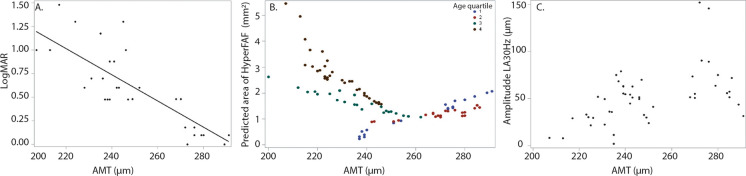
The average macula thickness is shown to decrease with worsening vision (**A**), increasing area of fundus hyperautofluorescence (**B**) and decreased amplitude of LA 30 Hz b-wave amplitude (**C**). In (**A**) the line represents the predicted value from GEE. In (**B**) and (**C**) each point represents a single eye at a single visit from one individual

Mean central macula thickness (CMT) was 184 ± 38 µm, and mean average macula thickness (AMT) was 246 ± 23 µm at first measurement (Table 2), and were both associated with better vision (β = 0.05 LogMAR per 10 µm, *p* =  < 0.0001; β = 0.1 LogMAR per 10 µm, *p* =  < 0.0001, Fig. 5A; respectively), even when correcting for age (Supplementary 5). Similarly, higher AMT was associated with smaller fundus hyperautofluorescence area (β = − 0.3 mm^2^ per 10 µm; *p* = 0.027; Fig. 5B), however this relationship was also shown to interact with age (*p* = 0.0002; Supplementary 5). To depict this interaction with age, the predicted area of fundus hyperautofluorescence was plotted against AMT with participants divided by age quartiles (Fig. 5B), illustrating that the change in area increases with age. Cone function was also associated with AMT, in which ffERG LA 30 Hz (Fig. 5C) and fused flicker (Supplementary 3A) b-wave amplitudes increased with higher AMT (β = 6.7 µV per 10 µm, *p* = 0.002; β = 2.7 µV per 10 µm, *p* =  < 0.0001; Table 3), but no association was found with peak time parameters (β = − 0.75 ms per 10 µm; *p* = 0.058; Table 3, Supplementary 3B). No association was found between AMT and pERG P50 (β = 0.003 per 10 µm; *p* = 0.96) and N95 (β = − 0.11 per 10 µm; *p* = 0.23) parameters (Table 3, Supplementary 3C and 3D).

**Fig. 6:**
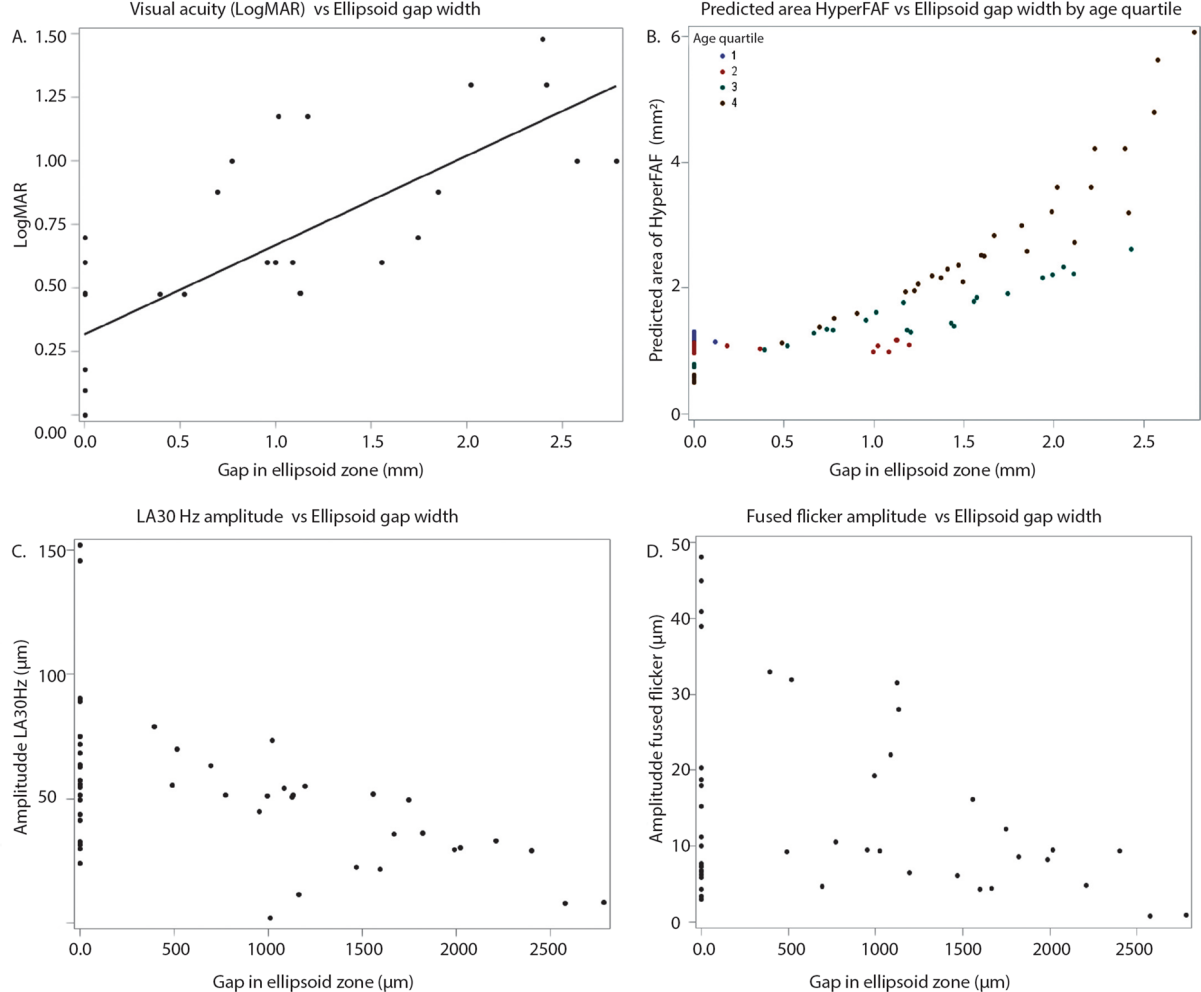
Lengthening of the disruption of the ellipsoid zone is associated with decreasing visual acuity (**A**), increasing area of fundus hyperautofluorescence (**B**), and decreasing amplitudes of both LA 30 Hz (**C**) and fused flicker (**D**) b-wave amplitudes. In (**A**) the line represents the predicted value from GEE. In (**B**), (**C**) and (**D**) each point represents a single eye at a single visit from one individual

Of the 32 eyes assessed, 18 (56%) displayed disruption of the EZ (Table 1). Mean EZ disruption length for all 32 eyes was 849 µm ± 903 µm at first measurement (Table 2). EZ disruption length was associated with decreasing visual acuity (β = 0.39 logMAR per 1000 µm; *p* =  < 0.0001; Fig. 6A), even when correcting for age (β = 0.24 logMAR per 1000 µm; *p* =  < 0.0001). Disruption of the EZ was associated with increased area of FAF (β = 1.1 mm^2^ per 1000 µm; *p* =  < 0.0001), however this relationship was shown to interact with age (*p* =  < 0.0001; Supplementary 5). To depict this interaction with age, the predicted area of fundus hyperautofluorescence was plotted against length of ellipsoid zone disruption with participants divided by age quartiles (Fig. 6B), illustrating again that the change in area increases with each year of age. Larger EZ disruption length was associated with decreased ffERG LA 30 Hz amplitude (β = − 19 µV per 1000 µm, *p* =  < 0.0002, Fig. 6C) and delayed peak time (β = 2.5 ms per 1000 µm, *p* = 0.0096; Supplementary 4A), as well as decreased fused flicker amplitude (β = − 4.4 µV per 1000 µm; *p* = 0.0028; Fig. 6D) (Table 3). The evidence for these relationships decreased when correcting for age, with only the delay in ffERG LA 30 Hz peak time remaining associated (β = 1.7 ms per 1000 µm, *p* = 0.049; Supplementary 5). No association was found between EZ and pERG P50 (β = − 0.01 µV per 1000 µm, *p* = 0.93) and N95 (β = 0.17 µV per 1000 µm, *p* = 0.49) parameters (Table 3, Supplementary 4B and 4C).

### Fundus autofluorescence

**Fig. 7:**
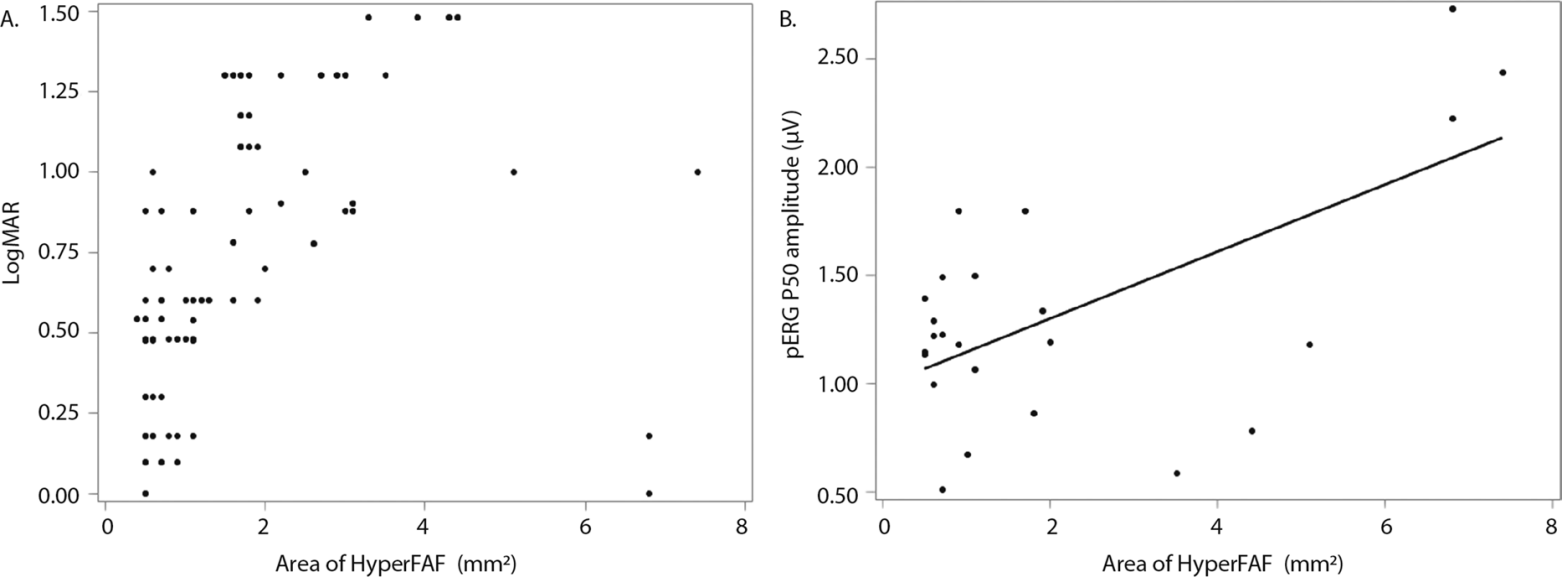
Relationship between area of fundus hyperautofluorescence and visual acuity (**A**) and pERG P50 at a 15 degree field (**B**). Each point represents a single eye at a single visit from one individual. In (**B**) the line represents the predicted value from the GEE

26 out of the 32 eyes assessed (81%) demonstrated fundal hyperautofluorescence at the first assessment (Table 1). The mean area of FAF at first measurement was 1.6 ± 1.5 mm^2^, with a range of 0–7.4 mm^2^ (Table 2). No association was found between FAF area and logMAR (β = 0.09 per 1 mm^2^; *p* = 0.087), however a trend is illustrated graphically in which patients with a larger area of hyperautofluorescence tended to have worse vision (Fig. 7A). No correlation was found between area of FAF and LA 30 Hz amplitude or peak time (β = − 3.3 µV per 1 mm^2^, *p* = 0.30; β = 0.6 ms per 1 mm^2^, *p* = 0.33), or fused flicker amplitude (β = 1.1 µV per 1 mm^2^; *p* = 0.52) (Table 3). Larger FAF area was associated with pERG P50 (β = 0.15 µV per 1 mm^2^; *p* = 0.0008; Fig. 7B), but not N95 (β = − 0.20 µV per 1 mm^2^; *p* = 0.097) (Table 3). These relationships increase in strength when correcting for age, in which N95 becomes more positive with increasing FAF area (P50: β = 0.18 µV per 1 mm^2^, *p* =  < 0.0001; N95: β = 1.9 µV per 1 mm^2^, *p* =  < 0.0001; Supplementary 5).

## Discussion

This retrospective cohort study describes the natural history of AD-CRD associated with mutations in the *GUCY2D* gene in 16 patients from an Australian cohort. Given the recently promising results of subretinal gene therapy in autosomal recessive GUCY2D-LCA [10], and the progress towards gene editing approaches to treat AD GUCY2D-CRD [11], reliable outcome measures of the slow disease progression of AD GUCY2D-CRD will be useful in designing future therapeutic interventions.

At first presentation with a mean age of 30 years, the visual acuity of 75% of the patients in this cohort was below the threshold for holding a driver’s licence in Australia [27], indicating the significant impact this disease can have on quality of life even in early adulthood. Over the period of observation, this vision decreased by 0.019 logMAR per year (*p* =  < 0.0001), which is similar to the recently reported European/United Kingdom cohort (0.022 logMAR [13] and 0.017 logMAR [12] per year). Despite the average length of observation of seven years, we only observed three patients that crossed the threshold defining legal blindness which made it difficult to accurately calculate an age at which patients with AD GUCY2D-CRD are expected to cross this threshold. This is a key threshold from both a functional perspective but also from the perspective of accessing services and assistance from public-funded resources [26]. The two recent studies in a similar cohort from Europe/United Kingdom determined that by close to 40 years of age, approximately 50% of patients with AD GUCY2D-CRD will be blind or have severe vision loss [12, 13], which is in keeping with the trends observed in this study. In this study we have mapped individual patient visual acuity with the course of their disease with many patients having multiple timepoints recorded. In addition, we have identified that a visual acuity level between LogMAR 0.5–0.6 is maybe another marker of impending accelerated visual decline adding another variable to determine the therapeutic window. A larger sample and longer follow up beginning from a younger age will assist in refining the therapeutic window.

Cone dysfunction as measured by ffERG was shown to worsen with age, and was correlated with decreased visual acuity, confirming the role of cone degeneration in the progression of this disease. Decreasing visual acuity and ffERG parameters were both associated with structural changes in the retina, including lengthening disruption of the EZ and reduced AMT, demonstrating a strong structure–function relationship in the progression of AD GUCY2D-CRD. The LA30Hz and FFAmp both showed similar relationships. This suggests that in *GUCY2D* retinopathy the FFAMP does not add additional information to that obtained by the ISCEV standard LA30Hz. These correlations also suggest that visual acuity is reliable as a key functional outcome marker of disease progression, which is in agreement with the recent proposal by Neubauer et al*.* [12] in which they propose visual acuity as the most relevant endpoint.

Previous studies have demonstrated changes in fundus autofluorescence associated with progression of disease [13, 28, 29], however ours is the first study to our knowledge that quantifies these changes over time in patients with GUCY2D-CRD. The structural changes detected as fundus hyperautofluorescence increased by 0.05 mm^2^ per year (*p* = 0.027), however no evidence of correlation was found between FAF and visual acuity, which is in keeping with previous findings in CRD populations [30]. There was a correlation between area of fundus hyperautofluorescence and pERG P50 representing retinal and macula dysfunction, however no association was identified with ffERG parameters, which is in contrast to previous studies of CRD populations [31]. This may suggest that the structural changes detected by FAF in GUCY2D-CRD follow a different course to that of the measured functional outcomes. In addition, although fundus hyperautofluorescence was correlated with ellipsoid zone disruption and macula thinning, these relationships were confounded by age. Indeed, 5 of the 16 patients demonstrated hyperautofluorescence without any disruption to the EZ, all of whom were below the mean age of 30 years on first visit. When the data was graphically depicted by age quartiles, those in the first and second quartile showed small areas of stable but present hyperautofluoresence with normal macula thickness and no or small changes in the EZ. Following this stable area, the slope of the curve changes to show a more linear relationship between increasing fundus hyperautofluorescence and deterioration of the ellipsoid zone and is dominated by those in the 3rd and 4th age quartiles. These findings suggest that UW-FAF may be useful in detecting early structural changes to the retina that precede other structural and functional deficits, and therefore may be a useful early structural biomarker to assist in developing a timeline of optimal therapeutic treatment. However, it must be noted that manual measurements used in this study are prone to error given the lack of distinct margins surrounding the area of hyperautofluorescence. A recent study using automated measurements of FAF suggests that quantifying FAF changes is more useful than OCT parameters in analysing visual field changes in patients with CRD [32]. Future studies would benefit from utilizing such automated method of detecting area and intensity of hyper/hypoautofluorescence to validate this as a useful structural biomarker.

It is noteworthy that significant variance in phenotype was demonstrated within families, and even between siblings. For example, of the brothers P2 and P3, at age 27 years P2 had a BCVA of 6/9 and no measurable EZ disruption, while P3 had a BCVA of 6/18 and EZ gap of 1.1 mm. (Fig. 4) Variable expressivity and incomplete penetrance has been demonstrated in previous studies [13, 22], and is likely multifactorial, however a clear mechanism determining phenotypic variability has yet to be established.

Limitations of this study include inherent variability in data availability and intervals between visits due to the retrospective nature of the study, as well as limited sample size. Future studies would benefit from a prospective method in which visits and data collection can be standardised across the sample. In addition, ideally participants would be studied from a young age, with length of observation over multiple decades to capture the time at which vision crosses important thresholds that impact the daily living of these patients, including ability to hold a driver’s licence and legal blindness. This study spanned on average seven years of observation, which provides one of the longest observation periods in this population, however the data most often did not cross these important thresholds. In any case, this study presents one of the few longitudinal studies with a large, multigenerational cohort of patients with GUCY2D-CRD, and the only study to quantify FAF changes over time in this population.

## Conclusion

In this study we have described the natural history of autosomal dominant GUCY2D-CRD in an Australian cohort using longitudinal data. Structural changes measured using OCT correlate with BCVA and ffERG parameters, highlighting their possible utility as reliable biomarkers for future therapeutic clinical trials. We have also highlighted the different rates of change in BCVA, EZ and FAF with disease duration which will assist in determining windows for therapeutic intervention.

This figure presents ffERG ISCEV standard responses, widefield fundus autofluorescence and macular OCT raster scans in a subset of the patient cohort compared to a normal subject. The cases chosen highlight the differences within families P1–P3, and with various ages P4, P6 and P15.

P1 illustrates normal scotopic (DA 0.01, DA 3.0 & DA 12.0) and reduced photopic (LA30Hz and LA 3.0) responses with a well-defined bulls eye pattern on FAF and loss of EZ line and outer retinal structures on OCT. P2 & P3 are the sons of P1. P2 has preserved scotopic responses and moderately reduced photopic responses. The FAF shows a blotchy central hyper autofluorescent pattern and OCT demonstrating blurring of the EZ line but no loss. P3 has normal scotopic and moderately reduced photopic ffERG responses. The FAF has an early bulls eye appearance with a bright hyperautofluorescent central region surrounded by ring of moderate hyperautofluorescence. The OCT has loss of the EZ line in parafoveal region corresponding to the boundary of the bulls eye macular hyperautofluorescence. P4 shows the normal scotopic and moderate reduction in photopic ffERG. FAF is diffuse blotch involving central fovea. The OCT shows early disruption of the EZ line. P6 similarly has normal scotopic ffERG and moderately reduced photopic ffERG. The FAF shows a compact bulls eye pattern of hyperautofluorescence. The OCT shows a relatively intact central subfoveal island and gaps in the EZ line in the parafoveal region. P15 is the oldest patient in the cohort the ffERG shows the normal scotopic but significantly reduced photopic ffERG. A well defined bulls eye pattern of fundus autofluorescence is show with rings of hyper and hypo autofluorescence. The OCT shows loss of the EZ line and outer retinal structures at the fovea.

ffERG full field electroretinogram, ISCEV International society for clinical electrophysiology of vision, DA dark adapted, LA light adapted, FAF fundus autofluorescence, OCT optical coherence tomography, EZ ellipsoid zone,

### Supplementary Information

Below is the link to the electronic supplementary material.

Supplementary file1 (JPG 1025 kb)

Supplementary file1 (JPG 1187 kb)

Supplementary file1 (JPG 922 kb)
