# Supplementary figures and images for: A natural history study of autosomal dominant GUCY2D-associated cone–rod dystrophy
Source: Doc Ophthalmol. 2023 Sep 29;147(3):189–201. doi: 10.1007/s10633-023-09954-7 (PMC10638150; doi:10.1007/s10633-023-09954-7)

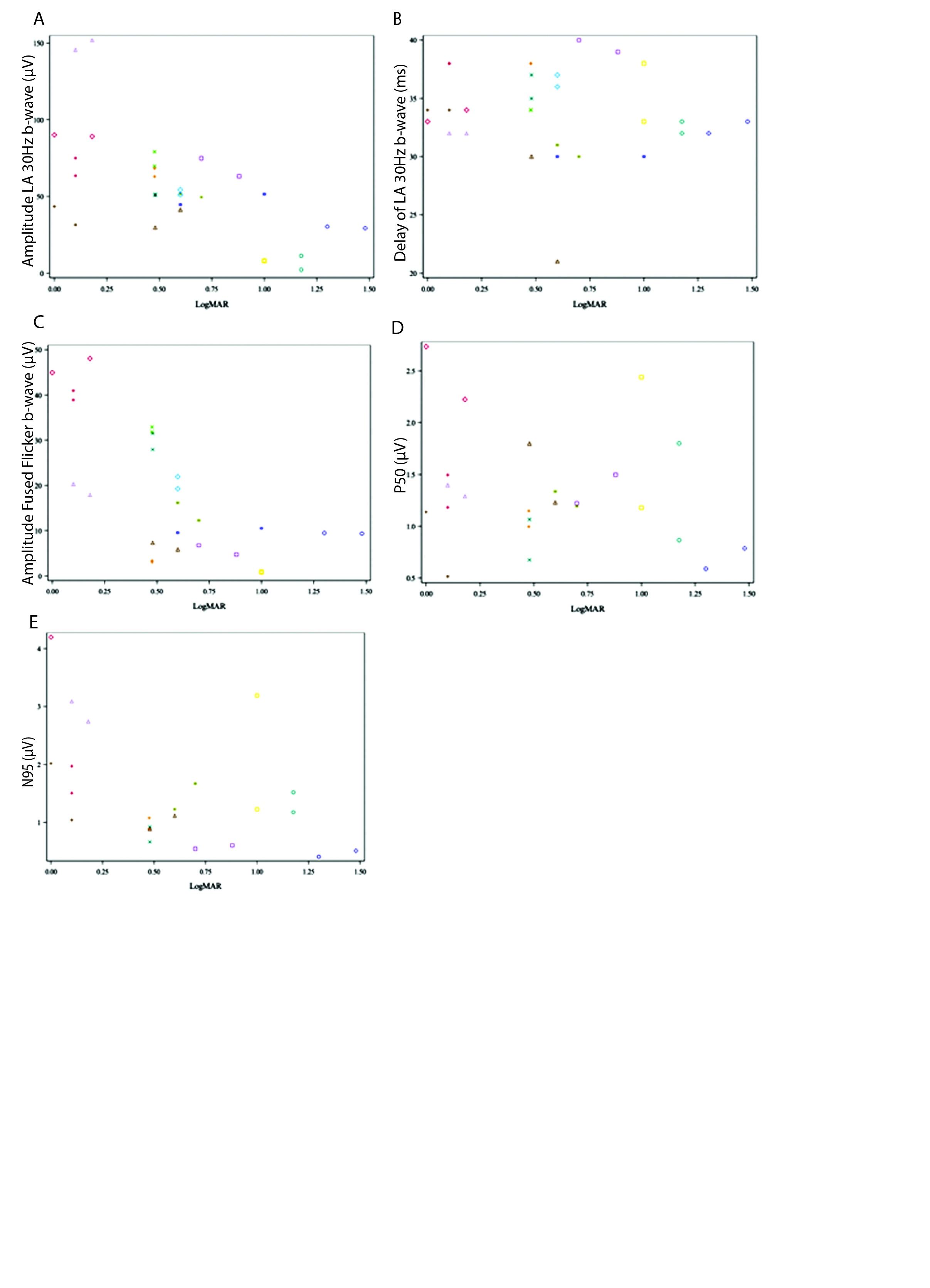

Supplement: Supplementary file 1 — Supplementary file1 (JPG 1025 kb) [file 10633_2023_9954_MOESM1_ESM.jpg]

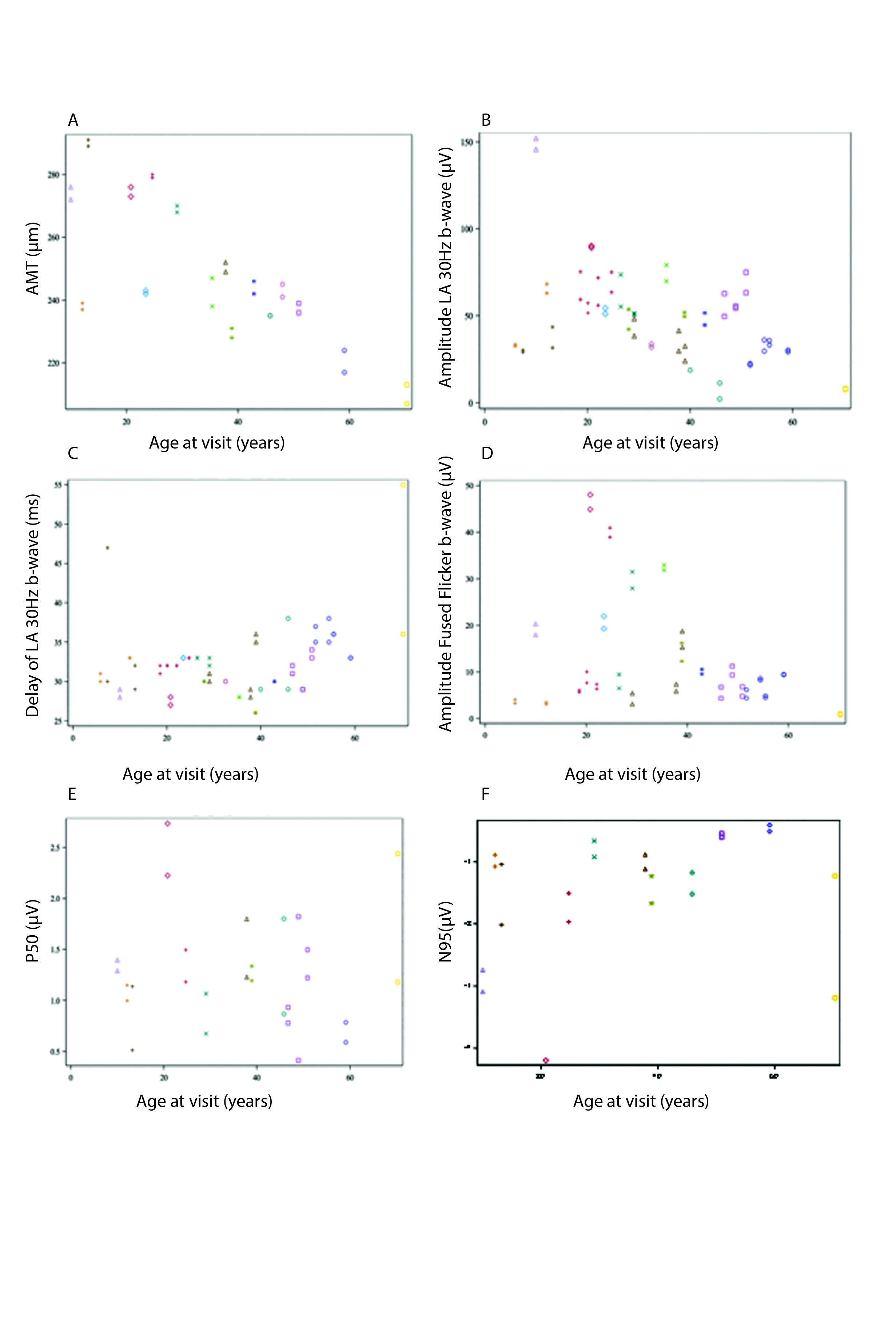

Supplement: Supplementary file 2 — Supplementary file1 (JPG 1187 kb) [file 10633_2023_9954_MOESM2_ESM.jpg]

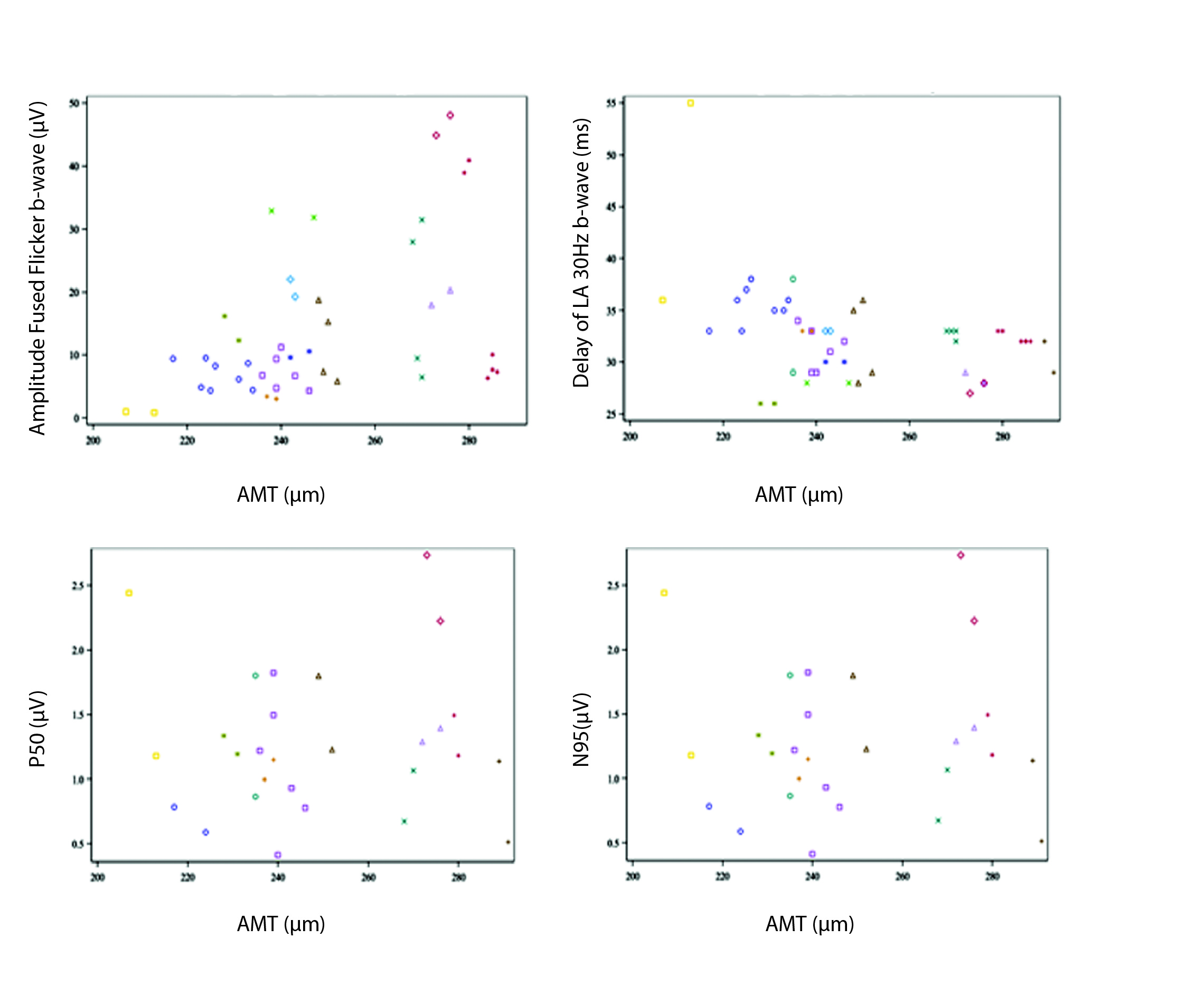

Supplement: Supplementary file 3 — Supplementary file1 (JPG 922 kb) [file 10633_2023_9954_MOESM3_ESM.jpg]
